# Geographical patterns of implementing a government subsidy program: implications for health outcomes and nutrient intake in Iran

**DOI:** 10.3389/fpubh.2024.1354099

**Published:** 2024-05-31

**Authors:** Mohammad Reza Pakravan-Charvadeh

**Affiliations:** Faculty of Agriculture, Lorestan University, Khorramabad, Lorestan, Iran

**Keywords:** nutrition policies, health economics, government subsidies, food price, human nutrition, Iran

## Abstract

**Introduction:**

The lack of access to a diverse and nutritious diet has significant health consequences worldwide. Governments have employed various policy mechanisms to ensure access, but their success varies.

**Method:**

In this study, the impact of changes in food assistance policy on food prices and nutrient security in different provinces of Iran, a sanctioned country, was investigated using statistical and econometric models.

**Results:**

Both the old and new policies were broad in scope, providing subsidized food or cash payments to the entire population. However, the implementation of these policies led to an increase in the market price of food items, resulting in a decline in the intake of essential nutrients. Particularly, the policy that shifted food assistance from commodity subsidies to direct cash payments reduced the price sensitivity of consumers. Consequently, the intake of key nutrients such as Vitamin C and Vitamin A, which are often constrained by their high prices, decreased. To improve the diets of marginalized populations, it is more effective to target subsidies towards specific nutrient groups and disadvantaged populations, with a particular focus on food groups that provide essential nutrients like Vitamin A and Vitamin C in rural areas of Iran.

**Discussion:**

More targeted food assistance policies, tailored to the specific context of each province and income level, are more likely to yield positive nutritional outcomes with minimal impact on food prices.

## 1 Introduction

A high-quality and nutritionally balanced diet is essential for health, well-being, learning, and workplace efficiency (1, 2). Achieving a healthy diet requires consuming a diverse range of food items (1, 3, 4). A balanced diet not only supplies the required energy for daily activities but also provides essential nutrients for growth and repair, thereby fostering strength and overall well-being (5). Merely focusing on calorie intake is insufficient to achieve a healthy diet (1). Half of the deaths related to diet are attributed to an improper balance of nutrients. Unhealthy diets are associated with the increasing prevalence of non-communicable diseases, such as obesity and overweight, globally (6, 7). Presently, the selection of food choices that impact balanced nutrition is predominantly influenced by factors such as price, convenience, taste, and health considerations (8). The impact of high food price inflation extends beyond macroeconomic stability and also affects small farmers and impoverished consumers in developing countries, where a significant portion of their income is allocated to food consumption (9). Food prices, especially those of fruits, vegetables, and proteins, strongly influence what is purchased and consumed, particularly among the poor (10). Food prices play a significant role in consumer food choices, which has implications for human health (11, 12). There is a clear association between food prices and consumer behavior (13, 14). To discourage unhealthy diets, various financial policies have been implemented worldwide (12, 15, 16). Developing a range of food assistance programs in different forms is a policy approach aimed at addressing the direct and indirect impacts of rising food prices on people’s health, particularly in developing countries (11, 17).

Governments often adopt food assistance policies as measures to enhance human capabilities (18). These policies, including direct and indirect subsidies, ensure that individuals with low incomes have access to healthy diets, leading to positive public health outcomes (15, 19, 20). Food assistance policies have been implemented in various settings and forms (11). Countries such as India, Bangladesh, Ethiopia, Zambia, Egypt, Algeria, Tunisia, and Iran, with different income levels and economic structures, have utilized non-targeted subsidies as food assistance programs to improve their residents’ nutrient quality and food security (15). However, most of these policies are non-targeted, as identifying eligible recipients requires accurate data and a proficient bureaucracy for implementation and oversight. Without such investments, the intended benefits may not reach impoverished households (11, 21). In developed countries, the situation is different. In the U.S., for example, the Supplemental Nutrition Assistance Program (SNAP) is a targeted policy designed to support low-income households (22, 23). Non-targeted reallocation of government subsidies (RGS) in Iran was formally launched on 18 December 2010. This policy involved phasing out public food and energy subsidies and replacing them with countrywide cash transfers (24). Under this policy, all individuals received an equal cash payment per month, regardless of their income level. This non-targeted approach differs from targeted policies that specifically assist low-income individuals. In Iran, the subsidy reform was initially well-received, as all citizens received the cash payment without any protests, unlike in countries such as Nigeria, Pakistan, Bolivia, and Indonesia, where the end of food subsidies led to citizen protests (11, 24). However, concerns have been raised regarding the impact of this reform on public health and the nutritional status of vulnerable households. Therefore, it is crucial to gain an evidence-based understanding of how this policy has affected the nutrition and health outcomes of households in different geographical locations.

Some studies have found that changes in government assistance for commodities precede significant increases in food prices (11, 25). Conducting an accurate evaluation of how demand responds to changes in prices and income resulting from the Reallocation of Government Subsidies (RGS) is crucial for making optimal policy decisions (26). Households residing in areas where food prices are already higher than the national average may face challenges in affording an adequate supply of nutritious food (22). Some studies have indicated that the price effect of implementing subsidy policies on nutrition, including calorie, protein, and fat intake, is negligible. However, these studies were limited in their consideration of only three nutrient categories (27). Another study has shown that the welfare of Iranian households decreases after implementing subsidy reforms, with urban households experiencing a greater decline compared to rural households. However, this study did not examine the nutrition and food security of Iranian households (11). Some scholars contended that implementing higher tax rates as a government policy is an effective strategy for reducing purchases or consumption. It has been observed that the impact of taxes on consumer behavior varies across income levels, with the lowest-income groups exhibiting the highest degree of responsiveness (28).

**Figure 1:**
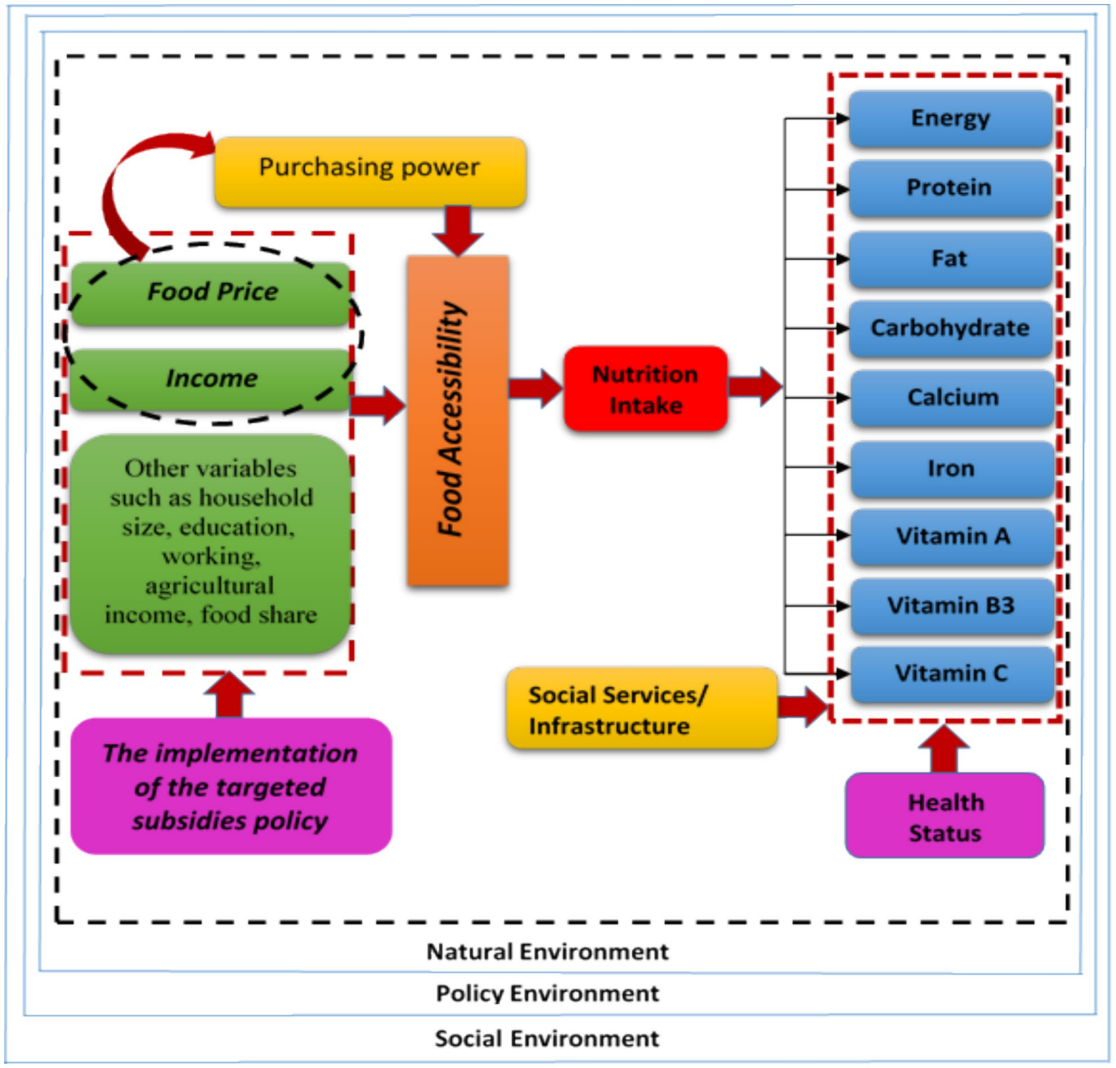
Conceptual relationship between the implementation of the targeted subsidies policy and the nutrient intake.

In this paper, we aim to bridge this knowledge gap by investigating the effects of a non-targeted food assistance program in Iran, known as the RGS, on *per capita* intake of calories, proteins, fats, carbohydrates, calcium, iron, vitamin A, vitamin B3, and vitamin C. This study holds global significance as it contributes to the understanding of the impact of government food assistance policies on nutrition outcomes, providing valuable insights that can inform policymakers not only in Iran but also in other countries facing similar challenges. By examining the effects of the Reallocation of Government Subsidies (RGS) program on nutrient intake and nutritional security, this study offers a framework for evaluating the effectiveness of non-targeted food assistance programs worldwide. Understanding the interplay between subsidy reforms, food prices, and nutrition outcomes is crucial for designing evidence-based policies that can improve the health and well-being of populations globally, particularly in the face of increasing prevalence of non-communicable diseases and rising food prices. Ultimately, the findings of this study can contribute to the development of more effective and targeted food assistance strategies that promote healthier diets and enhance public health on a global scale. Therefore, our objective is to assess the impact of Iran’s policies at the provincial level to determine which ones contributed to increased nutritional security. Figure 1 illustrates the potential mechanism through which the reallocation of government subsidies (RGS) can affect nutrient intake.

This figure depicts the hierarchical relationship between subsidy reform, food prices, and nutrition status, highlighting the impact of removing general subsidies for food and fuel on nutrition. What were the implications of reallocating the subsidies to provide direct fixed cash payments to all individuals, rather than indirect subsidies on commodities? How would the diagram differ if it analyzed the effects specifically for the poorer sectors of Iranian society? This study makes several contributions to the literature, including extracting the elasticities of all food groups through a partial equilibrium framework, considering geographical variations throughout Iran, empirically documenting the link among food prices, government subsidy reformation, and nutrition security, considering the interaction between the reform policy and nutrient outcomes simultaneously. Four questions will be addressed in this study. (1) How did food prices change over time and what is the interaction of price changes with implementing new assistance policies? (2) Did the RGS affect nutrient intake by surging the price of more nutritious food groups? (3) How did the intake of different food groups vary with variations in the prices of specific food commodities? (4) How did changes in nutrition intake after implementing RGS vary by socio-economic indicators?

### 1.1 Theoretical framework

To follow the objectives of the current study, we applied the following framework in three levels. At the micro-level, this study focuses on individual behavior and decision-making regarding food choices. This study emphasizes some factors such as price, convenience, taste, and health considerations that influence the selection of food items and overall nutrition. High food prices, especially for essential nutrients, can impact what individuals purchase and consume, particularly among low-income populations. Therefore, this study acknowledges the role of financial policies, such as subsidies and taxes, in shaping consumer behavior and promoting healthier diets. At the meso-level, this study examines the impact of government food assistance policies on nutrition outcomes and health at the provincial level in Iran. It considers the implementation of both old and new policies and their effects on food prices, nutrient security, and overall health outcomes. The study emphasizes the importance of targeting subsidies toward specific nutrient groups and disadvantaged populations, particularly in rural areas, to improve the diets of marginalized populations. At the macro-level, it focuses on the broader implications of government subsidy programs on public health and well-being. It recognizes that a high-quality and nutritionally balanced diet is essential for overall health, learning, and workplace efficiency. It acknowledges the association between unhealthy diets, non-communicable diseases, and rising food prices. It also highlights the role of food assistance policies in enhancing human capabilities and ensuring access to healthy diets, particularly for individuals with low incomes. Finally, this study emphasizes the importance of evidence-based policies that consider the interplay between subsidy reforms, food prices, and nutrition outcomes in addressing global challenges related to non-communicable diseases and increasing food prices.

## 2 Methods and materials

To analyze the data on food prices and household expenditure, we employed a generalized linear mixed model (GLMM) approach. In terms of the econometric model, demand equations were estimated for various nutrients, including calories, proteins, fats, carbohydrates, calcium, iron, vitamin A, vitamin B3, and vitamin C. This estimation was conducted using a Panel-Data approach spanning the years 1998 to 2020 (1). The specification of the model is theoretically linked to the health production function model proposed by Thomas (29), in which health or nutritional outcomes are considered as outputs that depend on multiple inputs (29). To estimate the elasticities of the key variables, we utilized Equation (1).


(1) $$\begin{array}{l} LnN_{ikt}=C_{0i}+\beta_{1i}RGS+\sum_{j=1}^{14}\alpha_{j}LnP_{jkt}+\sum_{j=1}^{14}\delta_{j}RGS\times LnP_{jkt}\\ +\beta_{2i}LnF+shar_{kt}\beta_{3i}RGS\times LnF^{shar_{kt}}+\beta_{4i}LnH^{\mathrm{Incom}e_{kt}}\\ +\beta_{5i}RGS\times LnH^{\mathrm{Inco}m_{kt}}+\beta_{6i}LnH^{Size_{kt}}+\beta_{7i}RGS\times LnH^{Size_{kt}}\\ +\beta_{8i}LnH^{Age_{kt}}+\beta_{9i}RGS\times LnH^{Age_{kt}}+\beta_{10i}LnH^{\mathrm{student}s_{kt}}\\ +\beta_{11i}RGS\times LnH^{\mathrm{student}s_{kt}}+\beta_{12i}LnH^{\mathrm{employe}e_{kt}}+\beta_{13i}RGS\\ \times LnH^{\mathrm{employe}e_{kt}}+\beta_{14i}LnH^{\mathrm{are}a_{kt}}+\beta_{15i}RGS\times LnH^{\mathrm{are}a_{kt}}+u_{\mathrm{it}}\\ \mathrm{In}\;\mathrm{which}\\ u_{\mathrm{it}}=\mu_{i}+v_{\mathrm{it}} \end{array}$$

The dependent variable is the logarithm of i^th^ nutrient consumed by k^th^ province in time *t*, RGS was coded as 0 for 1998–2020 when the policy had not been started and 1 for data gathered after the program was implemented, *LnP_jkt_* is the logarithm of the j^th^ food group price index in k^th^ province in time *t* (for 13 food groups), as shown in Table 1, *LogF^shar^* is the logarithm of food expenditure share of total monthly income, *LogH^Income^* is the logarithm of total household expenditure, *LogH^Size^* is the logarithm of household size, *LogH^Age^* is the logarithm of age of household head, *LnH^Students^* is the logarithm of the number of students in the household*, LogH^employee^* is the logarithm of the number of household members currently employed, and *LogH^area^* is the logarithm of home area (Square Meters) in k^th^ province in time *t*, and $u_{\mathrm{it}}$ the error term of i^th^ nutrient consumed in time t. Table 1 shows the summary statistics of selected household variables. The price indexes were estimated by Laspeyres method for 13 food groups in 30 provinces. Due to a large number of variables, fixed-effects models were used for these analyses, as there were insufficient degrees of freedom to fit province as a random effect.

**Table 1:** Definition and summary statistics of variables used in models.

Variable name	Variable definition
*RGS*	RGS is the time when the policy had not been initiated, 0 otherwise
*LnP_Cereal_*	Logarithm of Cereals Group Price Index
*LnP_V.Fat_*	Logarithm of Vegetable Fat Group Price Index
*LnP_A.Fat_*	Logarithm of Animal Fat Group Price Index
*LnP_R.Meat_*	Logarithm of Red Meat Group Price Index
*LnP_PU.Meat_*	Logarithm of Poultry Meat Group Price Index
*LnP_F.Meat_*	Logarithm of Fish Meat Group Price Index
*LnP_Legum_*	Logarithm of Legumes Group Price Index
*LnP_D.Fruit_*	Logarithm of Dried Fruit Group Price Index
*LnP_D.Prod_*	Logarithm of Dairy Products Price Index
*LnP_Fruit_*	Logarithm of Fruits Group Price Index
*LnP_Vegt_*	Logarithm of Vegetables Group Price Index
*LnP_Swee_*	Logarithm of Sweets Group Price Index
*LnP_Bevar_*	Logarithm of Beverage Group Price Index
*LnP_Spic_*	Logarithm of Spices Group Price Index
*LnFShar*	Logarithm of Household Food Share of Total Expenditure
*LnIncom*	Logarithm of Household Total Expenditure
*LnHSize*	Logarithm of Household Size
*LnHHAge*	Logarithm of Household Head Age
*LnNStud*	Logarithm of The Number of Students in Household
*LnNEmp*	Logarithm of The Number of employees in Household
*LnHHome*	Logarithm of Home Area (Square Meters)

To evaluate the effect of reallocation of the subsidies on food prices and nutrient intake, the elasticities of Equation (2) were used as below:


(2) $$\begin{array}{l} \Delta N_{i}=\overset{14}{\underset{j=1}{\sum }}\alpha_{j}\Delta P_{j}+\overset{14}{\underset{j=1}{\sum }}\delta_{j}RGS\times \Delta P_{j}\\ \;+\beta_{4}\Delta \mathrm{Income}+\beta_{5}RGS\times \Delta \mathrm{Income} \end{array}$$

ΔN is the percentage change of nutrient intake after the RGS, $\Delta P_{j}$ is the percentage change of the price of food group j, and ΔIncome is the percentage change of the household expenditures. If the difference between the effect of price and expenditure is positive, the policy led to increasing nutrient intake. If this difference has a negative sign, the policy has reduced nutrient intake. The impact of the RGS was estimated from the interaction effects between the coefficient for each variable and the RGS coefficient, which were different before (coefficient set as 0) and after imposition of the RGS. For instance, the effect of income before the RGS equals $\beta_{4}$ and after that was established it equals ( $\beta_{4}+\beta_{5})$ . The details of our sample construction are included in the next section.

## 3 Sample description

The data utilized in this analysis were obtained from the annual nationwide household food consumption survey conducted by the Consumer and Food Economics Statistical Center of Iran. The survey covered rural areas in each province simultaneously and included approximately 260,000 households across all 30 provinces. For this analysis, the data used were derived from usable food quantities, with food waste eliminated using waste coefficients suggested by the National Nutrition and Food Technology Research Institute. The data covered the period from 1998 to 2020.

To convert food quantities consumed at the household level into nutrition components, we employed the locally available food composition table. The quantities of nutrients consumed per household were calculated by multiplying the quantity of each food consumed by the household with the percentage of each nutrient available in each unit of food. These nutrient data were collected by the National Nutrient and Food Technology Research Institute of Iran in 2008. By summing up the respective nutrient quantities, we obtained an approximation of the amount of each nutrient available for consumption by the household every month.

To determine the nutrient intake per adult person per month, the estimated total nutrition was divided by the household size, using the method described by Gebre to account for variations in household member ages and consumption patterns (30, 31). Additionally, we calculated the average daily calorie intake per adult for each province. As individuals do not consciously purchase specific quantities of calories or other nutrients, but rather choose food groups based on taste and price, it is relevant to discuss the indirect demand for calories and other nutrients (32).

## 4 Results

During the period of analysis (1998–2020), price indexes were estimated for each food group. It was observed that the prices of all food groups increased, although not at a consistent rate. According to consumer behavioral theory, changes in food prices can impact food consumption patterns and nutrient intake over time (33).

The regression analysis examining the interaction between price changes over time and the RGS policy revealed a significant impact of the RGS on food price inflation. Specifically, prices demonstrated a faster rate of increase during the period when the policy was implemented, as indicated in Table 2. This can be observed by the steeper slope of the relationship and the lower intercept following the policy implementation, as described by Equation 1. Following the implementation of the RGS, which aimed to enhance households’ purchasing power through cash transfers, food prices generally experienced a more rapid increase. However, it is important to note that in many provinces, the implementation of the RGS counteracted, at least in the short term, an inflationary price surge that could have restricted access to a wide range of nutritious food options.

**Table 2:** The effect of the RGS on the intercept and slope of the food group price by provinces during 1998–2020.

*Variable*	*Cereal*	*Red meat*	*Chicken meat*	*Fish meat*	*Dried products*	*Animal fat*	*Vegetable oil*	*Fruits*	*Dried fruits*	*Vegetable*	*Legumes*	*Sweets*	*Spices*	*Beverage*
*Constant*	−267^***^	−184^***^	−161^***^	−186^***^	−185^***^	−192^***^	−233^***^	−167^***^	−199^***^	−179^***^	−219^***^	−189^***^	−159^***^	−188^***^
*Year*	0.134^***^	0.092^***^	0.081^***^	0.093^***^	0.093^***^	0.096^***^	0.117^***^	0.084^***^	0.100^***^	0.090^***^	0.110^***^	0.095^***^	0.080^***^	0.094^***^
*RGS (=0)*	17^***^	28^***^	28^***^	16^***^	32^***^	0.750	34^***^	2	56^***^	20^***^	14^***^	61^***^	−1.8	35^**^
Year*RGS (=0)	−0.009^***^	−0.013^***^	−0.014^***^	−0.008^***^	−0.016^***^	−0.0004	−0.017^***^	−0.001	−0.028^***^	−0.010^***^	−0.007^***^	−0.030^***^	0.001	−0.017^**^
*Province*														
*Ardebil*	0.014	0.003	−0.007	−0.020	−0.019	0.042	−0.027^*^	0.019^*^	−0.024	0.013	−0.001	−0.020	−0.043^***^	0.013
*Azghar*	−0.003	0.009	0.004	0.018	0.001	−0.014	−0.005	0.036^***^	−0.003	0.040^***^	0.005	−0.018	0.001	−0.038
*Azshar*	−0.015	0.013	−0.019^*^	0.008	−0.032^***^	−0.002	0.004	−0.022^**^	0.037^**^	−0.026^**^	−0.012	−0.016	0.014	0.010
*Bushehr*	0.035^**^	0.011	0.011	0.027^*^	0.007	0.016	0.049^***^	0.025^**^	0.009	0.006	0.031^**^	−0.073^***^	−0.002	−0.033
*Chaharmahal*	0.019	−0.002	0.013	0.049^***^	0.014	0.030	0.046^***^	−0.001	−0.023	0.008	−0.018	0.033^**^	−0.032^**^	0.017
*Esfahan*	0.047^***^	−0.019^**^	−0.002	−0.017	0.009	0.053^*^	0.016	0.002	0.010	−0.010	−0.011	0.014	0.003	0.032
*Fars*	0.057^***^	−0.006	0.017	0.020	0.000	0.086^***^	−0.022	−0.002	0.041^**^	−0.009	0.008	−0.027	−0.012	−0.007
*Guilan*	−0.011	0.020^**^	−0.008	−0.043^***^	−0.018	−0.029	−0.017	−0.001	0.028	−0.027^**^	−0.027^*^	0.004	0.020	0.100^**^
*Golestan*	−0.067^***^	−0.001	−0.006	−0.037^**^	−0.001	0.023	−0.035^**^	−0.001	0.040^**^	−0.012	0.026^*^	−0.049^***^	0.003	−0.013
*Hamedan*	0.022	0.007	0.002	0.098^***^	0.015	0.037	0.024	0.016	−0.068^***^	0.015	−0.001	0.021	0.025^*^	0.009
*Hormozgan*	0.004	0.027^***^	0.010	−0.021	0.024^*^	−0.075^***^	0.015	0.025^**^	−0.009	0.019^*^	0.024^*^	−0.029^*^	−0.001	0.069
*Ilam*	−0.006	−0.050^***^	0.036^***^	0.012	0.043^***^	−0.093^***^	−0.031^*^	0.021^*^	0.017	0.002	−0.014	−0.032^*^	−0.053^***^	−0.017
*Kerman*	−0.005	−0.029^***^	0.002	0.001	0.006	−0.127^***^	−0.005	−0.027^**^	−0.025	−0.003	0.006	−0.014	0.019	0.015
*Kermanshah*	0.007	−0.001	−0.007	0.031^*^	0.019	0.043	0.004	0.002	0.029	0.017	0.039^***^	0.020	0.006	−0.008
*KHjunubi*	0.026^*^	−0.018^*^	−0.015	−0.053^***^	−0.005	0.062^**^	−0.044^**^	−0.057^***^	−0.011	−0.006	0.010	0.011	0.026^*^	−0.003
*KHrazavi*	0.005	−0.010	−0.007	−0.007	−0.013	−0.003	−0.005	−0.015	0.000	−0.020^*^	−0.031^**^	0.016	−0.012	0.010
*KHshomali*	−0.038^**^	−0.018^*^	−0.004	−0.017	0.003	−0.006	0.018	−0.017	−0.015	−0.006	−0.020	0.029^*^	−0.006	0.012
*KHuzestan*	0.030^**^	0.021^**^	0.015	0.000	−0.009	0.126^***^	0.112^***^	0.028^**^	0.005	0.015	0.030^**^	0.074^***^	0.010	0.055
*Kohkiluye*	−0.011	−0.028^***^	0.014	0.047^***^	0.009	−0.013	−0.005	−0.010	−0.024	−0.012	0.000	−0.004	−0.006	−0.139^***^
*Kurdestan*	−0.012	0.018^*^	0.001	−0.027^*^	0.015	−0.002	−0.018	0.022^**^	0.002	0.012	0.015	0.009	−0.030^**^	−0.102^**^
*Lorestan*	−0.014	−0.004	0.007	−0.063^***^	−0.029^**^	−0.003	0.056^***^	0.007	−0.036^*^	0.011	−0.021	0.038^**^	−0.024^*^	−0.095^*^
*Markazi*	−0.004	−0.011	0.003	0.013	0.007	0.028	−0.018	−0.029^***^	0.023	−0.012	−0.003	−0.036^**^	−0.033^**^	0.030
*Mazandaran*	−0.005	−0.002	−0.025^**^	−0.020	0.000	0.023	−0.037^**^	0.015	0.037^**^	−0.037^***^	0.026^*^	−0.035^**^	0.002	0.034
*Qazvin*	−0.006	0.018^*^	−0.008	0.006	−0.012	−0.006	−0.014	−0.007	−0.010	−0.004	−0.015	−0.042^**^	−0.003	−0.106^**^
*Qom*	−0.013	0.000	−0.016	−0.024	−0.029^**^	0.002	0.033^*^	−0.025^**^	−0.030^*^	−0.012	−0.004	0.096^***^	0.024^*^	−0.002
*Semnan*	0.028^*^	0.006	0.008	0.004	0.007	−0.037	0.041^**^	−0.013	−0.006	0.002	−0.001	0.028^*^	0.012	0.013
*Sistan*	−0.014	−0.009	−0.009	−0.051^***^	−0.005	−0.161^***^	−0.087^***^	−0.023^**^	0.024	−0.018^*^	−0.001	−0.085^***^	0.014	−0.001
*Tehran*	−0.069^***^	0.051^***^	−0.002	0.017	0.001	−0.014	−0.005	0.035^***^	−0.007	0.057^***^	0.007	0.024	0.021	0.004
*Yazd*	0.033^**^	−0.008	0.000	0.033^**^	−0.023^*^	0.001	−0.044^**^	0.004	−0.026	−0.004	−0.033^**^	0.041^**^	0.009	0.055

^*^, ^**^, ^***^ represent levels of significance at 10, 5, and 1%, respectively.

The analysis further reveals variations in nutrient prices across different provinces. The coefficients presented in Table 2 indicate the effects on the regression intercepts for the price-time relationships. For instance, in Tehran, the heavily populated capital city of Iran, red meat and fruit had the highest prices. This is likely because Tehran relies heavily on food imports from other provinces since it does not produce most of its food locally. Conversely, Mazandaran province, known for its chicken meat production, had the lowest coefficient for chicken meat prices in Table 2. This suggests that chicken meat is more affordable in Mazandaran compared to other provinces.

The regression analysis presented in Table 3 demonstrates that the RGS policy had a significant inverse effect on the intercept and a non-significantly positive impact on the slope of nutrient intake. This indicates that although nutrient intake declined over time compared to the period before the implementation of the RGS, the decline was slower after the policy was introduced. Further investigation into the province effect on the nutrient intake model, as shown in Table 3, revealed that certain provinces such as Ardebil, Azerbaijan Gharbi (Azghar), Azerbaijan sharqi (Azshar), Mazandaran, Kurdistan, and Kermanshah had higher carbohydrate intake compared to other provinces. This can be attributed to the higher production and consumption of cereals in these provinces, which are rich in carbohydrates and starch. Before estimating the model, the presence of multicollinearity among all independent variables was examined using the variance inflation factor (VIF) test. The results indicated that there was no significant correlation among these variables. Following the regression analysis, an econometric partial equilibrium model (Equation 1) was employed to delve deeper into the results, as shown in Table 4. The first stage estimation revealed that the explanatory variables accounted for up to 90% of the nutritional status. Durbin-Watson and Jarque-Bera’s statistics indicated that none of the assumptions of the models (normality of residuals, homoscedasticity, and residual autocorrelation) were violated. In terms of the coefficient of the RGS, it exhibited a higher value in the functions describing the intakes of vitamin A and vitamin C, while a lower value was observed for protein. This suggests that during the policy years, the greatest impact was related to vitamin A and vitamin C intake.

**Table 3:** The effect of the RGS on the intercept and slope of the nutrient intake by provinces during 1998–2020.

*Variable*	*Calorie*	*Protein*	*Fat*	*Carbohydrate*	*Calcium*	*Iron*	*Vitamin A*	*Vitamin B3*	*Vitamin C*
*Constant*	35^***^	36^***^	22^***^	40^***^	47^***^	25^***^	30^***^	26^***^	17^**^
*Year*	−0.015^***^	−0.017^***^	−0.010^***^	−0.018^***^	−0.022^***^	−0.012^***^	−0.014^***^	−0.012^***^	−0.008^**^
*RGS (=0)*	28^***^	24^***^	35^***^	35^***^	34^***^	21^***^	34^***^	35^***^	16^**^
Year*RGS (=0)	−0.014^***^	−0.012^***^	−0.018^***^	−0.017^***^	−0.017^***^	−0.010^***^	−0.017^***^	−0.017^***^	−0.008^**^
*Province*									
*ardebil*	0.157^***^	0.137^***^	0.098^***^	0.153^***^	0.129***	0.097^***^	0.075^***^	0.135^***^	0.061^***^
*azghar*	0.125^***^	0.095^***^	0.105^***^	0.126^***^	0.084^***^	0.103^***^	0.051^**^	0.124^***^	0.110^***^
*azshar*	0.063^***^	0.039^**^	0.023	0.053^***^	0.040^**^	0.028^*^	0.061^***^	0.080^***^	0.002
*bushehr*	−0.082^***^	−0.013	−0.104^***^	−0.028	−0.020	0.023	0.097^***^	0.044^***^	0.100^***^
*chaharmahal*	−0.040^**^	0.048^***^	−0.115^***^	0.022	0.080^***^	−0.005	0.014	−0.071^***^	−0.032
*esfahan*	−0.006	0.018	0.018	−0.008	0.049^***^	0.035^**^	0.153^***^	0.002	0.058^***^
*fars*	0.029^*^	−0.019	0.041^**^	−0.015	0.005	0.008	0.097^***^	−0.079^***^	0.105^***^
*guilan*	−0.044^**^	−0.004	0.000	−0.003	−0.025	−0.025	0.035^*^	0.037^**^	0.142^***^
*golestan*	−0.104^***^	−0.090^***^	−0.024	−0.078^***^	−0.134^***^	−0.155^***^	−0.184^***^	−0.041^**^	−0.136^***^
*hamedan*	0.059^***^	−0.036^**^	−0.004	0.001	−0.009	−0.021	0.019	−0.025	−0.016
*hormozgan*	−0.126^***^	−0.040^**^	−0.102^***^	−0.093^***^	−0.061^***^	−0.033^*^	−0.038^*^	−0.080^***^	−0.063^***^
*ilam*	0.074^***^	0.012	0.015	0.014	0.010	0.025	0.003	−0.012	−0.014
*kerman*	0.049^***^	0.029^*^	−0.010	0.023	0.009	0.011	−0.177^***^	−0.033^**^	−0.126^***^
*kermanshah*	0.102^***^	0.030^*^	0.058^***^	0.066^***^	0.029^*^	0.027	−0.055^***^	−0.018	0.003
*khjunubi*	0.030^*^	0.007	0.070^***^	−0.012	0.031^*^	0.000	−0.059^***^	−0.091^***^	−0.121^***^
*khrazavi*	0.024	−0.025	0.073	−0.024	−0.022	−0.010	−0.027	0.005	−0.084^***^
*khshomali*	−0.029^*^	−0.037^*^	−0.013	−0.006	−0.060^***^	−0.058^***^	−0.148^***^	0.011	−0.127^***^
*khuzestan*	−0.001	−0.004	−0.020	−0.014	0.005	0.004	−0.017	−0.107^***^	0.055^**^
*kohkiluye*	−0.152^***^	−0.090^***^	−0.152^***^	−0.110^***^	−0.088^***^	−0.069^***^	−0.085^***^	−0.135^***^	0.016
*kurdestan*	0.045^**^	0.047^***^	0.090^***^	0.091^***^	0.053^***^	0.048^***^	0.033^*^	0.105^***^	0.040^*^
*lorestan*	−0.021	0.029^*^	−0.019	0.023	0.006	0.008	−0.042^**^	0.068^***^	−0.074^***^
*markazi*	0.025	−0.004	0.024	−0.009	0.035^**^	0.022	0.114^***^	0.025	0.055^**^
*mazandaran*	−0.052^***^	0.013	−0.001	−0.019	0.029^*^	0.010	0.146^***^	0.027	0.204^***^
*qazvin*	0.101^***^	0.026	0.103^***^	0.039^**^	0.042^**^	0.042^**^	0.118^***^	0.025	0.106^***^
*qom*	−0.072^***^	−0.014	−0.041^**^	−0.033^*^	−0.015	0.022	0.015	0.084^***^	−0.064^***^
*semnan*	0.006	−0.009	0.100^***^	0.005	−0.027	−0.003	−0.055^***^	0.039^**^	−0.007
*sistan*	−0.073^***^	−0.034^**^	−0.113^***^	−0.058^***^	−0.097^***^	−0.051^***^	−0.377^***^	−0.177^***^	−0.198^***^
*tehran*	−0.097^***^	−0.146^***^	−0.064^***^	−0.154^***^	−0.097^***^	−0.135^***^	0.069^***^	−0.117^***^	0.020
*yazd*	−0.024	−0.033^*^	−0.068^***^	−0.046^**^	−0.042^**^	−0.028^*^	0.078^***^	0.009	−0.076^***^

The dependent variable is the logarithm of the nutrient intake.

^*^, ^**^, ^***^ represent levels of significance at 10, 5, and 1%, respectively.

**Table 4:** The effect of the RGS on nutrition intake in Iran rural area.

Variables	Energy	Protein	Fat	Carbohy…	Calcium	Iron	Vitamin A	Vitamin B3	Vitamin C
C	−0.75	−2.71^***^	−1.55^*^	−1.50	−2.95^***^	−4.53^***^	0.69	−0.94	−3.36^*^
RGS (=1)	−148^*^	−87^***^	−168^**^	−188^**^	−155^***^	−101^***^	−585^***^	−168^*^	−426^***^
RGS*Year (=1)	0.074^*^	0.044^***^	0.083^*^	0.094^**^	0.078^***^	0.051^***^	0.291^***^	0.084^*^	0.212^***^
Year	−0.183^**^	−0.048^***^	−0.132^*^	−0.226^**^	−0.007	−0.034^***^	−0.694^***^	−0.325^**^	−0.454^***^
Log P(Cereal)	−0.140^*^	−0.224^***^	0.017	−0.209^*^	−0.072	−0.265^***^	0.101^***^	−0.198^***^	0.121^**^
Log P (Red Meat)	−0.020	−0.156^***^	−0.091	−0.043	−0.171^*^	−0.216^*^	0.112^**^	−0.121^**^	0.114
Log P (Poultry Meat)	−0.193	−0.247	−0.513^***^	−0.209^*^	−0.205^***^	−0.135	−0.058	−0.199^***^	0.106^***^
Log P (Fish Meat)	0.019^***^	−0.109^***^	0.164^*^	0.029	0.022	0.152^**^	−0.251^**^	−0.003^**^	−0.069
Log P (Fruits)	−0.095	0.380^***^	−0.310^***^	−0.041	−0.255^***^	−0.152^*^	−0.524^*^	−0.088	−0.406^***^
Log P (Vegetable)	−0.232^**^	−0.235^***^	−0.011	−0.389^*^	0.189^***^	−0.251^***^	−0.385^**^	−0.233^**^	−0.446^***^
Log P (Dried Fruits)	0.038	−0.150^***^	−0.007	−0.067^**^	−0.199^***^	−0.185^***^	−0.173^*^	−0.118^**^	0.111
Log P (Dairy Products)	0.068	−0.017^**^	−0.011	0.162	−0.159^**^	0.052	0.434	−0.106^***^	−0.169^**^
Log P (Vegetable Oil)	0.197^**^	0.069^***^	−0.018	0.199^***^	0.100^***^	0.164^***^	−0.057	0.123^*^	−0.036^*^
Log P (Animal Fat)	0.030	−0.026	−0.031^***^	0.020	0.027	−0.024	0.128^***^	0.011	−0.038^**^
Log P (Legumes)	−0.190^***^	−0.042^**^	−0.079	0.240^***^	−0.072^*^	0.054	0.041	- 0.183^**^	0.085^*^
Log P (Sweet)	−0.225^***^	−0.226^***^	−0.182^***^	−0.208^***^	−0.143^***^	−0.141^***^	−0.002	−0.057	0.039
Log P (Spices)	−0.242^**^	−0.179^*^	−0.059^**^	−0.116^*^	0.090	−0.169^*^	−0.010^*^	−0.182^**^	−0.162^*^
Log P (Beverage)	0.033^***^	0.012^*^	0.043^***^	0.032^***^	0.003	0.024^***^	0.045^***^	0.027^**^	0.038^***^
Log HH Size	−0.451^**^	−0.146	−0.552^***^	−0.253	−0.176	−0.333^**^	−0.947^**^	−0.616^**^	−0.985^***^
Log HH Student	−0.212^**^	−0.234^***^	−0.158^***^	−0.210^**^	−0.132^*^	−0.216^***^	−0.028	−0.070	−0.005
Log HH Age	0.575^**^	0.141	−0.035	0.400^***^	0.614^***^	0.485^**^	−0.302^*^	0.537^**^	−0.406^**^
Log HH Employee	−0.018	−0.119^***^	0.032	−0.039	0.051^*^	−0.151^***^	−0.008	−0.067	0.088
Log HH Income	0.629^***^	0.674^***^	0.684^***^	0.596^***^	0.679^***^	0.766^***^	0.722^***^	0.726^***^	0.688^***^
Log HH Food Share	0.637^***^	0.917^***^	0.686^***^	0.557^***^	1.059^***^	0.900^***^	1.104^***^	0.512^***^	1.081^***^
RGS*Log P (Cereal)	−0.033	0.213^***^	−0.035^***^	−0.015	0.040	0.301^***^	−0.405^***^	0.089	−0.090^***^
RGS*Log P (Animal Fat)	−0.043	0.009	−0.049	−0.010	0.017	−0.018	−0.164^***^	−0.024	−0.082^***^
RGS*Log P (Red Meat)	0.079	−0.157^**^	0.163^*^	0.180	0.132	−0.120^**^	−0.810^***^	0.118	−0.136^***^
RGS*Log P (Poultry Meat)	0.225	0.199	−0.082^***^	0.128	0.297^**^	0.325^**^	−0.381	0.030	−0.124^***^
RGS*Log P (Fish Meat)	−0.010	−0.204^***^	−0.090	−0.076	−0.165^*^	−0.017^**^	0.319^**^	0.086	0.310^*^
RGS*Log P (Fruits)	0.020	0.229^***^	0.289^*^	−0.004	−0.246^*^	−0.015^**^	0.199	0.122^**^	0.210
RGS*Log P(Vegetable)	−0.101^**^	0.253^**^	0.016^*^	−0.162	0.108	−0.103^***^	0.577^***^	−0.117^**^	−0.149^***^
RGS*Log P (Dried Fruits)	−0.102^*^	0.021	0.032^**^	−0.181^***^	0.086^**^	−0.065^**^	0.367^***^	−0.172^**^	−0.072
RGS*Log P (Dairy Products)	−0.145	0.232	−0.072	0.003	−0.106^***^	−0.048	−0.637^**^	0.077	−0.083
RGS*Log P (Vegetable Oil)	−0.213^***^	−0.350^***^	−0.118^**^	−0.328^***^	−0.058^***^	−0.147^***^	0.335^***^	−0.101^***^	0.006
RGS*Log P (Legumes)	−0.105	−0.114^**^	−0.097^**^	−0.041	0.170^***^	0.050	0.140	0.164^**^	0.100
RGS*Log P (Sweet)	0.238^**^	0.297^***^	−0.094^***^	0.297^***^	0.275^**^	0.232^***^	0.149^**^	0.151^*^	0.240^*^
RGS*Log P (Spices)	0.185	−0.026	0.182^**^	0.001	−0.158^**^	0.031	0.430^***^	0.031	0.188^*^
RGS*Log P (Beverage)	0.167^**^	0.027	−0.093^**^	0.150^*^	0.100^**^	−0.129^***^	−0.004	0.254^***^	−0.069
RGS*Log HH Size	0.069	0.155	0.134^*^	0.029	0.022	0.022	−0.108	0.223	−0.178
RGS*Log HH Student	−0.058	−0.151	0.062	−0.181^***^	−0.079	−0.027	−0.120	−0.234^**^	0.171^***^
RGS*Log HH Age	0.015	−0.597^**^	0.598^*^	−0.469^**^	−0.257	−0.340^*^	0.180	−0.680^**^	0.432^*^
RGS*Log HH Home	−0.181^**^	0.043	−0.067	−0.143^**^	−0.025	0.022	0.049	−0.054	0.022
RGS*Log HH Food Share	0.326^**^	0.098	0.203	0.359^***^	−0.071	0.130	0.124	0.258^*^	0.100
RGS*Log HH Income	0.148^**^	0.048	0.029	0.148^**^	−0.011	0.037	0.040	−0.004	−0.039

The dependent variable is the logarithm of the nutrient intake.

^*^, ^**^, ^***^ represent levels of significance at 10, 5, and 1%, respectively.

The coefficients in Table 4 do not suggest that nutrient intake increased due to the negative effects of food prices on nutrient functions. Rather, they indicate that the rate of reduction in nutrient intake over time was slower compared to the period before the implementation of the RGS policy. The intake equations presented in Table 4 demonstrate how the intake of different nutrients varies in response to changes in food commodity prices. The coefficients of the food commodity price elasticities offer insights into the complex relationship between price and nutrition. As expected, most of these coefficients are negative, indicating that as prices rise over time, nutrient intake tends to decline. For instance, an increase in cereal prices leads to a reduction in energy intake but an increase in the intake of Vitamin A and Vitamin C. This can be attributed to the relatively larger inflation in cereal prices compared to other nutrient groups, as shown in Table 2. As a result, consumption shifts from cereals to fruits and vegetables, which experience less price inflation. The presence of the RGS policy modifies these interdependencies, as indicated by the significant deviations from zero for the RGS*price interactions. This effect is particularly pronounced for certain groups, such as the impact of the price of sweets combined with the change in purchasing power resulting from the RGS. This significantly affects the intake of most nutrients. In other words, the cash provided by the RGS enables additional expenditure on sweets, leading to an increased proportion of intake from nutrient-poor but calorie-dense foods. Overall, the relationship between price and nutrient intake is complex, with the RGS policy influencing these dynamics by altering consumption patterns and the affordability of different food items.

Some scholars found an increase in the price of sweets in both low-income and high-income countries was associated with reduced consumption of sweets and increased consumption of all other food items, except fats and oils (14). Regarding the impact of red meat prices, the study revealed negative and significant effects on protein, calcium, iron, and vitamin B3 intake. However, it was positively and significantly associated with vitamin A intake. The price of poultry meat had negative and significant effects on fat intake (*p* < 1%), carbohydrate intake (*p* < 10%), calcium intake (*p* < 1%), and vitamin B3 intake (*p* < 1%). Conversely, it had a positive effect on vitamin C intake (*p* < 5%). Following the implementation of the RGS policy, the coefficient of the poultry meat price change caused a shift in calcium intake from negative to positive. Analyzing fish meat prices (Table 4), it was observed that after the RGS, an increase in fish prices had a significant negative effect on calcium and protein intake. As for vegetable oil, after the price increase due to the RGS, it hurt energy intake, protein intake, fat intake, and carbohydrate intake, while it had a positive and significant effect on calcium intake, iron intake, vitamin A intake, and vitamin B3 intake. However, its effect on vitamin C intake was found to be insignificant. After the policy implementation, the coefficient for vitamin C remained negative, while the coefficient for vitamin A became negative. This suggests that vegetable oil was substituted for animal fat after the RGS. The price of dried fruits had a negative and significant effect on all nutrient groups, except for three. Similarly, the price of fruits had a significantly negative effect on vitamin C intake, vitamin A intake, iron intake, calcium intake, and fat intake. Before the RGS, the increase in spice prices led to a decrease in nutrient intake, except for calcium (insignificant). However, after the RGS, the price of spices had a significant effect only on fat intake, vitamin A intake, and vitamin C intake, indicating a change in the relationship between spices and other foods. Lastly, the share of household expenditures on food had a positive effect on all nutrient functions, implying that as the share of household expenditures allocated to food increased, nutrient intake also increased.

After the implementation of the RGS policy, a 1% increase in all prices and household incomes resulted in a decrease in the intake of various nutrients. Specifically, there was a decrease in calorie intake by 0.018%, protein intake by 0.036%, fat intake by 0.041%, carbohydrate intake by 0.045%, calcium intake by 0.041%, iron intake by 0.003%, vitamin A intake by 0.074%, vitamin B3 intake by 0.053%, and vitamin C intake by 0.069%. Furthermore, the analysis of socioeconomic determinants revealed certain associations with nutrient intake. The size of the household and the number of students were found to be significantly and inversely related to some nutrient intakes. In other words, larger households and households with more students tended to have lower intakes of certain nutrients. On the other hand, the age of the household head had a significantly positive effect on all nutrient functions, except for vitamin A and vitamin C. This means that as the age of the household head increased, nutrient intake tended to be higher across various nutrients, except for vitamin A and vitamin C. These findings highlight the influence of prices, household income, and socio-economic factors on nutrient intake, providing insights into the complex dynamics of food consumption and nutrition.

The study demonstrates that the RGS policy had a positive impact on nutrition by mitigating the effects of price changes and maintaining nutrient intake, despite a period of rapid price inflation. The magnitude of price inflation differed across food groups, and the RGS helped attenuate the negative impact on nutrient intake. Among the nutrients, the greatest decrease after the RGS was observed in vitamin A intake, with a reduction of 0.074%. Following vitamin, A, vitamin C showed the second-largest decline. Fruits and vegetables are known to be excellent sources of vitamin C, and their intake may have been affected by the changes in prices. These findings underscore the importance of considering the specific nutrient content of different food groups and their affordability when assessing the impact of price changes on nutrition. The RGS policy played a crucial role in preserving nutrient intake, particularly for vitamin A and vitamin C, despite the challenges posed by the period of rapid price inflation.

## 5 Discussion

After the implementation of the RGS, which involved non-targeted income transfers to adults that could be used for any household expenditure, food prices generally experienced a relatively faster increase compared to the pre-policy period. Among food groups, sweets, and dried fruits showed the largest price increases. By estimating price elasticities and analyzing nutrient intakes, the study revealed that after the RGS, price changes had a lesser impact on nutrient intake compared to before the policy was introduced (34). Despite the period of rising prices, the RGS provided increased purchasing power. However, its effect on nutrient intake was complex, as the purchasing patterns of different food items changed in response to price fluctuations, resulting in dietary changes. It is important to note that the impact of the RGS policy varied across nutrient groups, and provincial factors played a significant role. Geographical variation emerged as one of the key factors that need to be considered when formulating or modifying policies. These findings highlight the need to consider regional factors and the diverse effects of policies on different nutrient groups. Understanding and addressing geographical variations can contribute to the development of more effective policies aimed at improving nutrition and addressing food price dynamics (22, 35).

The price elasticity of cereal, fish meat, legumes, and spices groups did not change for energy intake after the implementation of the RGS. However, the elasticity of sweets became positive, indicating that as food prices increased, people tended to preferentially purchase sweets. This shift toward consuming more energy-dense but nutrient-poor food items with high sugar content, combined with reduced physical activity, has implications for increased rates of obesity (36). Sweet foodstuffs and beverages, which have a strong hedonic appeal, particularly among children and young people (37), have been identified as potential contributors to the obesity epidemic not only in Iran but globally, especially in developing countries (38, 39). Dried fruits play a significant role in household diets as they are important sources of calcium, vitamin A, and antioxidants. Before the RGS, dried fruits had a more positive effect on calcium, vitamin A, and protein intake. However, after the policy implementation, a 1% increase in the price of dried fruits resulted in a decrease in energy intake by 0.102%, carbohydrate intake by 0.248%, calcium intake by 0.113%, iron intake by 0.250%, and vitamin B3 intake by 0.290%. Increasing fruit prices also led to a reduction in nutrient intake. Fruits are essential sources of vital nutrients such as dietary fiber, potassium, vitamin C, and folate, which are often under-consumed. After the RGS, the impact of rising fruit prices had a stronger effect on the coefficients of overall calcium intake (reduction of 0.501) and iron intake (reduction of 0.167).

After the implementation of the RGS, increases in household income did not have a significant effect on nutrient intake, except for energy and carbohydrate intake. It is important to consume sweets and fats sparingly due to their high caloric content. Sweets are not recommended as a fundamental food group, and their consumption should be limited due to their chronic health effects (40). While the increment in household income resulting from the RGS policy provided households with sufficient purchasing power, it did not lead to a significant improvement in food and nutrition security (41). The policy-induced income increases only had a positive and significant impact on carbohydrate and energy intake. While calorie intake was maintained, nutrient security was not ensured. Although the RGS was implemented during a period of global food price volatility, the counterfactual scenario of “what would intake rates have been if the RGS had not increased purchasing power?” might have resulted in even worse health outcomes. Therefore, the policy potentially prevented a more detrimental situation in terms of health. However, it is worth noting that the RGS led to increased consumption of sweets, as indicated by the interaction coefficient between the policy and sweet consumption. This higher consumption of sweets contributes to the rise in obesity and overweight cases in Iran (42). In Iran, the rates of obesity, as measured by the BMI index, NCHC, and percentile above 95, were 17.4, 7.6, and 7.4%, respectively. Obesity in Iran is twice the global average. The increased consumption of sweets due to the policy may pose a public health risk. These findings emphasize the need for comprehensive measures to address the potential negative impacts of increased sweet consumption and the associated rise in obesity rates. Efforts to promote healthier dietary choices and combat obesity should be considered alongside income-related policies to ensure improved overall health outcomes.

After the implementation of the RGS, the price increase in the vegetables and fruits group had a greater negative effect on vitamin C compared to other food groups. Vitamin C deficiency can lead to scurvy, a potentially life-threatening condition. The antioxidant properties of vitamin C also help stabilize folate in food and plasma. Increased excretion of oxidized folate derivatives can contribute to the incidence of scurvy in humans (43). The results of the study confirmed a negative association between household size and nutrition intake. As households grow in size, the constraints on food budgets reduce the accessibility of adequate nutrition. While some studies have found a positive relationship between household size and food security, those studies did not specifically focus on nutrition security. Food security may have been maintained due to an increase in household members participating in the labor force (44, 45). Other studies have found an inverse relationship between household size and food security (46, 47). The age of the household head has shown a positive association with nutrient intake in various studies (48, 49). Older household heads may exhibit more conservative food choices, which can have a positive impact on nutrient intake. The increase in the age of the household head is likely linked to greater experience and knowledge of nutrient needs (47, 50). After the RGS, the age of the household head was significantly and positively associated with the consumption of fat, iron, and vitamin C. The share of food expenditure also had a significant direct impact on all nutrient intakes.

Providing subsidies to all adults to support healthier eating during a period of rapid price inflation had a range of positive and negative effects due to changing purchasing habits, some of which were unexpected and had unexpected impacts on dietary nutrient intake. Nevertheless, despite impressive economic growth and increasing per-capita income in recent decades in Iran, households still face nutrient deficiencies. The study found that the RGS had the greatest negative effect on vitamin A, followed by vitamin C intake. For the non-meat animal products group (milk, cheese, and eggs), price increases had the most detrimental effect on vitamin A intake, particularly among low-expenditure households.

### 5.1 Strengths and limitations of the study

Among the economic and policy models that focus on the impact of food assistance programs on food security and nutrition, some studies (46, 51), have only examined the effect of price and income changes on calorie intake at a country level. In our analysis, we investigated the effect of these changes on nutrient intake at the provincial level. For instance, some scholars have used food group elasticity at the national level to study the impact of food price increases on nutrient intake during a recession (46). Our study’s geographic specificity is novel, and we presented a comprehensive and theoretically consistent analytical framework to support its usefulness. While some studies have attempted to explore the direct and indirect effects of food price shocks using yearly data and information on nutrition and malnutrition (1, 26, 46), most of them have relied on inadequate data from specific regions or countries and have not utilized large datasets. In our study, we analyzed the impact of food price shocks through the implementation of a government subsidy policy shift, with a focus on geographical patterns. Certain studies argue that obtaining precise quantitative measures of dietary intake is time-consuming and requires a significant number of nutritional experts to create extensive food composition databases for data analysis and collection (11). However, in our study, nutrient intake was calculated with minimal errors. It is crucial to develop a model that allows for the investigation of interaction effects resulting from policy implementation. In contrast to studies that have solely evaluated the impact of new assistance programs in Iran through inter-sectoral analysis of food consumption and household welfare (11), our study meticulously examined the effect on household nutrient intake.

This study does share some limitations with other studies. Firstly, calculating household nutrient intake using large datasets was time-consuming and complex, making human errors inevitable. Secondly, due to the size of the dataset used in this study, there may be biases in the results, as demonstrated by other studies (52).

### 5.2 Conclusion and policy implications

The policy that governs the distribution of subsidies holds significant importance for impoverished households. In Iran, the current practice of providing direct payments to all individuals without considering their specific needs has led to inappropriate and detrimental consequences in terms of food access for the poor. Our research strongly suggests that further investigation is needed in order to address these issues. Allocating subsidies specifically to impoverished households would not only help preserve government financial resources but also improve nutrition security. This study utilized the best available micro-sectoral data in Iran to analyze the impact of the implemented RGS on nutrient intake across distinct areas of the country. The differential rates of commodity price inflation and changes in food group consumption in various provinces have created a complex relationship between prices and nutrient intake, yielding both positive and negative effects. For instance, increased cereal prices resulted in higher vitamin intake through reduced cereal consumption and increased vegetable consumption, which was an unforeseen effect for non-economists, particularly those outside of the US and Europe. Economists who solely rely on static single market/commodity approaches would not have been able to predict this with the precision achieved through our combination of sound economic theory, robust data, and sophisticated econometric modeling. To mitigate the undesirable effects of the RGS, it would be beneficial to implement a policy that controls and reduces fluctuations in food prices, particularly for foods that are vital in addressing vitamin deficiency. To increase vitamin A consumption, which is highly sensitive to price increases, “food grants” could be provided to impoverished households based on their income and expenditure. These grants could include dairy products and other commodities rich in different sources of vitamin A. Additionally, the government could implement an educational program to raise awareness among households about the importance of vitamin A and vitamin C and provide guidance on various methods to maintain an adequate intake. Policymakers and stakeholders should reevaluate socio-economic factors, such as household size, number of students, and geographical location, in order to develop a new nutrition policy that improves the nutrient status of the population. The impact of the RGS on nutrient intake varied significantly from one province to another, highlighting the necessity of formulating geographically targeted policies rather than implementing a uniform policy for the entire country. Implementing a program similar to the Supplemental Nutrition Assistance Program (SNAP) in the United States to compensate for reduced nutrition intake is imperative. As demonstrated by our results, nutrition policies should consider geographical variations and socioeconomic factors to identify areas where households suffer from nutrient deficiencies.

### 5.3 Recommendation for the future studies

In this study, we utilized a generalized linear mixed model approach to examine the impact of the RGS on health outcomes, using nutrient intake as an indicator of overall health status. Although we didn’t employ sophisticated techniques, such as a computable general equilibrium (CGE) model, to comprehensively analyze all aspects of implementing the RGS in Iran, our findings strongly suggest that future studies should consider the geographical variations in the implementation of the RGS within the country to develop and recommend appropriate policies. While a partial equilibrium analysis may seem straightforward and practical for evaluating the effects of a specific policy on health outcomes and nutrition intake, this approach may yield inaccurate results by neglecting inter-sectoral relationships (11). To obtain more precise and appropriate findings, employing a general equilibrium framework would be advantageous. This would allow for a comprehensive assessment of the complex interactions and feedback effects among different sectors and the economy as a whole.

## Data availability statement

The raw data supporting the conclusions of this article will be made available by the authors, without undue reservation.

## Author contributions

MP-C: Conceptualization, Data curation, Formal analysis, Investigation, Methodology, Project administration, Resources, Software, Supervision, Validation, Writing – original draft, Writing – review & editing.
